# Static and Dynamic Mechanical Properties of 3D-Printable Aligner Resins: An In Vitro Study with FTIR Chemical Characterization

**DOI:** 10.3390/jfb17070340

**Published:** 2026-07-14

**Authors:** Marco Serafin, Elisa Boccalari, Marina Borgese, Alberto Caprioglio, Mario Raspanti, Gilberto Binda, Piero Antonio Zecca

**Affiliations:** 1 Department of Biomedical, Surgical and Dental Sciences, University of Milan, 20122 Milan, Italy; elisa.boccalari@unimi.it (E.B.); alberto.caprioglio@unimi.it (A.C.); 2 Department of Medicine and Innovative Technology, University of Insubria, 21000 Varese, Italy; marina.borgese@uninsubria.it (M.B.); mario.raspanti@uninsubria.it (M.R.); pieroantonio.zecca@uninsubria.it (P.A.Z.); 3 IRCCS Fondazione Ca’ Granda, Ospedale Maggiore Policlinico, 20122 Milan, Italy; 4 Department of Science and High Technology, University of Insubria, 22100 Como, Italy; gilberto.binda@uninsubria.it; 5 Norwegian Institute for Water Research, 0001 Oslo, Norway

**Keywords:** 3D printable resins, FTIR spectroscopy, mechanical properties, orthodontic aligners, three-point bending test

## Abstract

Background: Directly printed aligners are advancing rapidly, but the mechanical behavior of the resins behind them is still only partly understood. This in vitro study compared the static flexural behavior, short-term stress relaxation, and FTIR profiles of five Class IIa-certified 3D-printable resins for direct orthodontic aligners. Methods: The five resins, TC-85, TA-28, DCA, Clear-A V2, and Ortho Flex, were printed as standardized rectangular bars and tested at 37 °C. Three-point bending to 1 mm deflection yielded the maximum flexural stress and the flexural modulus, while a 30 min hold at fixed deflection captured stress relaxation. FTIR added a qualitative chemical characterization. Results: Differences between resins were substantial. DCA led on every static measure, pairing the highest flexural stress and modulus with the highest final relaxation modulus and the best stiffness retention. Clear-A V2 was also statically stiff but retained force only intermediately, whereas TC-85 combined high stiffness with pronounced relaxation. Ortho Flex performed modestly under static loading yet held on to a moderate fraction of its stiffness, and TA-28 relaxed the most. Conclusions: Directly printed aligner resins are mechanically heterogeneous, and static bending alone did not predict short-term force stability. Relaxation metrics should therefore accompany static testing whenever a resin is selected for a specific clinical purpose.

## 1. Introduction

Patient demand for aesthetic, removable alternatives to fixed appliances has made clear aligner therapy one of the fastest-growing areas of contemporary orthodontics [1]. For most of that history, aligners have been produced by thermoforming polymeric foils over dental models. Additive manufacturing is now changing this picture, allowing clear aligners to be printed directly from photopolymer resins and offering potential gains in workflow simplification, appliance reproducibility, thickness control, and design customization [2].

How an aligner behaves mechanically depends on both what it is made of and how it is made. Direct printing adds a further layer of process-dependent variables to this equation, from printer technology and build orientation to layer thickness, resin-specific exposure settings, and post-curing protocols. Earlier work has confirmed that the mechanical properties of 3D-printed aligners can shift with the printing system and the chosen manufacturing parameters, even if the size and clinical weight of these effects still vary from one material and protocol to another [3,4].

Biomechanically, a good aligner material has to strike a balance between stiffness, flexibility, elastic recovery, and dimensional stability [5,6]. Too much elasticity can blunt force delivery and compromise tracking, while too much rigidity makes insertion, adaptation, and everyday comfort worse. Complicating matters further, these materials are viscoelastic, so the force they exert after insertion tends to fade over time under constant deformation at mouth temperature [7,8]. Static flexural parameters, on their own, therefore give only a partial picture, and time-dependent properties deserve to be measured under standardized conditions that approximate the oral environment.

Thermoformed aligner materials have been studied in considerable depth. Thermoforming itself, along with thermal exposure, water storage, simulated aging, and the intraoral environment, can alter the thickness, stiffness, flexural properties, and force transmission of thermoplastic foils [7,9,10,11]. The clinical message is clear: aligner performance is shaped not only by the nominal material but also by everything the foil goes through during manufacturing and aging.

The evidence base for printable aligner resins, by contrast, is still thin. A handful of studies have looked at individual printable or shape-memory resins, at the role of printing orientation and thickness, and at 3D-printed versus thermoformed comparisons [12,13,14,15]. What remains rare is a head-to-head evaluation of several commercially available Class IIa-certified resins under a single, temperature-controlled static and stress-relaxation protocol. Without such comparisons, it is hard to tell apart materials that are merely stiff at first loading from those that can also hold their stiffness under sustained deformation.

Most printable aligner resins are vat-photopolymerized urethane- and methacrylate-based systems produced by digital light processing or LCD stereolithography, and a few have built-in shape-memory behavior [2,14]. Because the exact composition of commercial resins is proprietary and largely undisclosed, chemical characterization becomes a useful way to make sense of material-dependent mechanical differences. Fourier-transform infrared (FTIR) spectroscopy offers a non-destructive way to read the major functional-group patterns and compare spectral fingerprints across polymeric materials. In its qualitative form, it cannot quantify the degree of conversion, residual monomer content, or crosslink density, but it can flag material-specific chemical heterogeneity and help frame the interpretation of structure–property trends.

Against this background, the primary aim of the present in vitro study was to compare, under standardized conditions at 37 °C, the static flexural behavior and short-term stress-relaxation performance of five commercially available Class IIa-certified resins for directly printed aligners. Its novelty lies in bringing all five resins together within a single temperature-controlled static and stress-relaxation protocol, a comparison which has not previously been reported and one that separates materials that are merely stiff at initial loading from those that also keep their stiffness under sustained deformation. Attenuated total reflectance FTIR played only a supporting role, providing qualitative characterization to aid interpretation rather than serving as a study endpoint.

## 2. Materials and Methods

This in vitro study examined the mechanical and chemical characteristics of commercially available 3D-printable resins intended for the direct fabrication of orthodontic aligners. Five Class IIa-certified, aligner-specific materials were selected: Ortho Flex (NextDent, Soesterberg, The Netherlands), Clear-A V2 (Senertek, Karataş, Turkey), DCA (LuxCreo, Chicago, IL, USA), TA-28 (Graphy, Seoul, Republic of Korea), and TC-85 (Graphy, Seoul, Republic of Korea). The Graphy resins (TC-85 and TA-28) and the LuxCreo resin (DCA) were supplied directly by their respective manufacturers.

Rectangular bar specimens measuring 25 mm × 5 mm × 0.5 mm were designed digitally and printed with resin-specific parameters and the post-processing protocol recommended by each manufacturer. Every specimen was built in a 90° (vertical) orientation, chosen to reproduce the loading direction of the vestibular wall of a clinical aligner. The 0.5 mm thickness and overall geometry were likewise selected to approximate the wall of a real aligner, so that the derived parameters describe a clinically representative shape rather than a standard test coupon. For this reason the testing geometry and calculations were adapted from, rather than made fully compliant with, the three-point bending principles of ISO 178 [16] for thin rectangular specimens. Printing was carried out on a AccuFab-CEL open-system LCD printer (Shining3D, Hangzhou, China; 405 nm) at a 100 µm layer thickness in a climate-controlled room at 22 °C and 50% relative humidity. The manufacturer-validated resins (Graphy TC-85 and TA-28, and NextDent Ortho Flex) were run on the printer’s locked resin profiles, in which the per-layer exposure is fixed by the manufacturer and cannot be accessed by the user; the open-system resins (LuxCreo DCA and Senertek Clear-A V2) were printed with a normal-layer exposure within the manufacturer-recommended range (approximately 2–3.5 s) and a bottom-layer exposure of 10–30 s. Each resin was then post-processed according to its own manufacturer’s protocol. The Graphy resins (TC-85 and TA-28) were centrifuged (Tera Harz Spinner) to remove residual resin and UV post-cured for 20 min under a 95% nitrogen atmosphere in the Tera Harz Cure THC2 (Graphy, Seul, Republic of Korea), then rinsed in hot water. LuxCreo DCA was ultrasonically washed in isopropyl alcohol, heat-cured for 10 min at 130 °C, and UV post-cured at 405 nm for 20 min, in accordance with the manufacturer’s protocol. Clear-A V2 was cleaned by water centrifugation and post-cured for 10 min in the glycerin-immersed Curie Cure unit (Senertek) without a nitrogen atmosphere, then rinsed in hot water and briefly in boiling water. NextDent resin was cleaned in ethanol (>90%) and post-cured in the LC-3DPrint Box (NextDent) for 30 min at a chamber temperature of at least 60 °C, in line with the manufacturer’s instructions for use.

Dimensional accuracy was checked with a digital caliper of 0.01 mm resolution, and any specimen showing visible defects or dimensional deviations was discarded. Five valid specimens (*n* = 5) per resin were carried forward to the mechanical tests.

### 2.1. Static Three-Point Bending Testing

**Figure 1:**
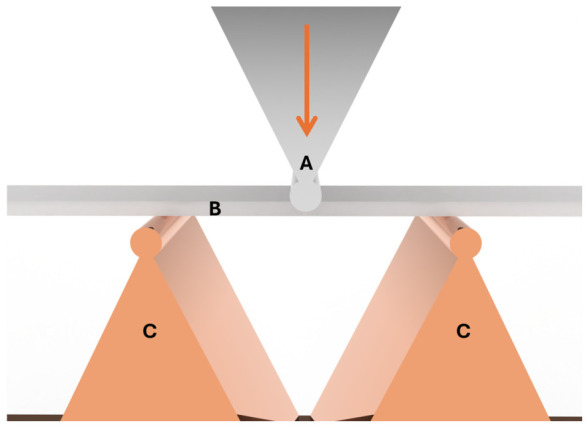
Schematic of the three-point bending setup. A: descending loading nose (the arrow indicates the load direction); B: specimen; C: the two cylindrical supports connected to the load cell. The distance from the loading nose “A” to each support “C” is 5 mm, giving a support span (C to C) of 10 mm.

Static flexural behavior was measured with a custom three-point bending device built by the Department of Physics specifically for low-load testing of orthodontic polymeric specimens. The apparatus brought together two parallel cylindrical supports, a centrally aligned loading nose, a calibrated low-capacity load cell, and a motorized, displacement-controlled actuator (Figure 1). Before testing began, the force-measuring chain was calibrated across the expected working range with traceable reference loads, and displacement accuracy was verified against a calibrated dial indicator. At the start of each test the load cell was zeroed and then checked by placing a known 100 g mass on its sensitive surface; only a reading of 100 g allowed the test to proceed, and any discrepancy triggered repeat zeroing and calibration. Loads were consequently recorded in grams-force and converted to Newtons for the stress and modulus calculations. The support span, loading-nose alignment, and specimen dimensions were re-checked with calibrated instruments before every session. The 10 mm support span, combined with the 0.5 mm specimen thickness, gave a span-to-thickness ratio of 20:1, higher than the 16:1 specified by ISO 178 [16] and therefore further limiting the contribution of transverse shear to the measured deflection. Finally, specimens were equilibrated in water at 37 °C for 24 h and tested under thermostatic conditions at 37 °C.

The device was validated by checking force linearity, displacement accuracy, repeatability, and zero drift, and by benchmarking it against a calibrated universal testing machine under identical conditions. Matched specimens from the same production batch were run on both systems using the same support span, crosshead speed, and environment. The calibration check confirmed excellent metrological performance: repeated measurements of the 100 g reference mass gave a mean of 100.03 ± 0.18 g (coefficient of variation, 0.18%), a negligible mean bias of +0.03 g on Bland–Altman analysis (95% limits of agreement, −0.32 to +0.38 g), and an intraclass correlation coefficient indicating excellent reliability (ICC = 0.999). Overall, the procedure amounted to a modified three-point bending protocol grounded in ISO 178 [16] and ASTM D790, with every deviation from the standard reported explicitly.

A vertical load was applied at mid-span at a crosshead speed of 1 mm/min until the specimen reached 1 mm of deflection. This displacement was split into 20 increments of 0.05 mm, with force–displacement data logged continuously. Because the test ended at a predefined displacement of 1 mm rather than at fracture (the materials being viscoelastic), the maximum stress was taken as the maximum flexural stress within that 1 mm window rather than as a fracture strength.

Let F be the load, δ the mid-span deflection, L the support span, b the specimen width, h the specimen thickness, and V the specimen volume. Outer-fiber stress and mid-span strain were calculated as:

σ = 3FL/2bh^2^

ε = 6hδ/L^2^

Therefore, the following static parameters were derived:

- Flexural strength, *σ_f_*_max_ = 3*F_max_ L*/2*bh*^2^. It corresponds to the maximum outer-fiber stress reached within the imposed displacement window (δ_max_ = 1 mm; not necessarily fracture).
- Flexural modulus, *E_f_* = *L*^3^*m*/4*bh*^3^ with *m* on the full 0–1 mm loading window. This value is reported as an apparent flexural modulus over the 0–1 mm loading window and is used as a comparative index of bending stiffness rather than as a strictly linear-elastic modulus.

### 2.2. Stress-Relaxation Testing

Short-term viscoelastic behavior was captured through stress-relaxation testing at fixed deformation. Each specimen was deflected to 1 mm at mid-span and held there for 30 min at 37 °C while the decay in load was recorded over time. At every time point, the relaxation modulus was obtained from the beam relation for three-point bending under fixed deflection:

*E*(*t*) = *F*(*t*)*L*^3^/4*bh*^3^*δ_hold_*

where F(t) is the load at time *t* and δ_hold_ is the fixed deflection of 1 mm.

The following stress-relaxation parameters were derived:

- Initial relaxation modulus, *E*_0_ = *E*(*t* = 0^+^). The instantaneous (unrelaxed) modulus at the start of the hold characterizes the glassy/instant elastic response.
- Final relaxation modulus, E_30_ = E(t = 30 min). Equilibrium (relaxed) modulus at the end of the hold; reflects long-term viscoelastic compliance.
- Percent relaxation, %Relax = 100 × (E_0_ − E_30_)/E_0_. The fractional decrease in modulus during the hold; higher values indicate stronger viscoelastic behavior (softening).
- Relaxation ratio, RR = *E*_30_/*E*_0_. A dimensionless stiffness-retention index.

Residual central force after 30 min was estimated from the relaxation modulus using:

*F* = 4*bh*^3^*δ_E_*/*L*^3^

For the present geometry, this reduces to F [N] = 0.0025 × E [MPa]. These force values were used only as geometry-specific comparative estimates and were not interpreted as direct clinical orthodontic forces.

### 2.3. FTIR Spectroscopy

Chemical characterization relied on ATR-FTIR spectroscopy. Spectra were collected with a diamond ATR module across the mid-infrared range from 4000 to 650 cm^−1^ at a spectral resolution of 4 cm^−1^, averaging 32 scans per measurement under ambient laboratory conditions. The specimens analyzed were post-cured pieces identical to those used for mechanical testing, examined without any extra surface preparation, and a background spectrum was taken between samples to keep instrumental drift in check.

Each resin was run in triplicate. The spectra were smoothed with a Savitzky–Golay filter, normalized to the maximum absorbance peak, and averaged across the three replicates for qualitative interpretation. We inspected the major functional-group regions, including bands linked to urethane, methacrylate, and acrylate moieties, paying particular attention to the fingerprint region between 1250 and 750 cm^−1^, where material-specific differences in co-monomer structure and network chemistry tend to appear. Throughout, the FTIR data were treated qualitatively and were never used to infer the degree of conversion or crosslink density.

### 2.4. Statistical Analysis

Data are given as mean ± standard deviation (SD) for the five specimens tested per resin (*n* = 5). The distribution of each endpoint within a resin was checked with the Shapiro–Wilk test. Given the small group size (*n* = 5), between-resin differences were assessed with the non-parametric Kruskal–Wallis test, followed by Dunn’s post hoc pairwise comparisons with Holm correction for multiple testing, and a two-sided *p* value below 0.05 was taken as statistically significant. Reproducibility was good: the within-resin coefficient of variation was about 3.6% for the static endpoints, 2.3% to 2.4% for the relaxation moduli, and below 0.2% for the percentage relaxation and the relaxation ratio. Using five specimens per resin is consistent with common practice in in vitro mechanical testing of dental polymers and with our pilot feasibility work; the combination of low within-group variability and large between-resin separation made this sample adequate for detecting the sizeable material-dependent differences of interest, even if it was never meant to resolve fine distinctions between closely ranked resins. All analyses were run in Python 3.11 (SciPy and scikit-posthocs).

## 3. Results

Three-point bending and stress-relaxation testing revealed substantial differences among the directly printed aligner resins. Tellingly, static flexural performance and short-term viscoelastic stability did not line up in the same order, so a resin that absorbed a lot of work or felt stiff at first did not necessarily hold its force best under constant deformation.

### 3.1. Static Mechanical Properties

Table 1 summarizes the static three-point bending results. Since every specimen was loaded to a predefined 1 mm deflection rather than to fracture, the stress endpoint should be read as the maximum flexural stress reached within that imposed displacement window.

At 1 mm deflection, DCA reached the highest maximum flexural stress, ahead of Clear-A V2 and TC-85, while TA-28 and Ortho Flex sat at the bottom of the range. The apparent flexural modulus followed much the same order, with DCA stiffest and Clear-A V2, TC-85, Ortho Flex, and TA-28 trailing behind. Dunn post hoc comparisons (Holm-adjusted) confirmed significant gaps between the compliant resins (Ortho Flex and TA-28) and the stiffest resin (DCA) for both maximum flexural stress and apparent flexural modulus (*p* < 0.05), and also between Ortho Flex and Clear-A V2; adjacently ranked resins, however, did not differ significantly at this sample size.

### 3.2. Stress-Relaxation Properties

Under a fixed 1 mm mid-span deflection, the relaxation modulus decayed at a material-specific rate over the 30 min observation window (Table 2). DCA started highest and finished highest after 30 min, and it also held onto the largest fraction of its initial stiffness.

Clear-A V2 and Ortho Flex formed an intermediate dynamic group, though they got there differently. Clear-A V2 kept a higher absolute final modulus, and therefore more residual stiffness in absolute terms. Ortho Flex, on the other hand, posted a slightly higher relaxation ratio, meaning that it preserved a better relative share of its stiffness despite starting from a lower initial modulus.

The steepest time-dependent decay belonged to TC-85 and TA-28. TA-28 in particular ended with the lowest final modulus, the highest percentage relaxation, and the lowest relaxation ratio, marking both resins as prone to pronounced short-term viscoelastic softening under constant deformation. For the relaxation endpoints, post hoc comparisons cleanly separated DCA from the compliant resins at the extremes of the distribution (*p* < 0.05), even though not every adjacently ranked pair reached significance.

**Figure 2:**
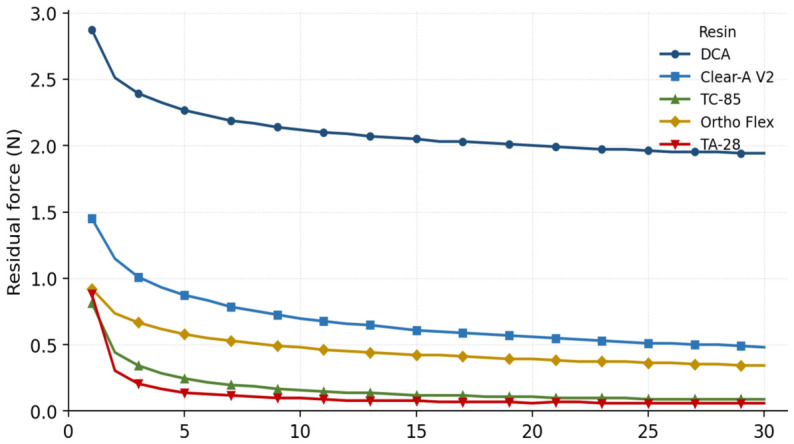
Residual force retention (N) under fixed deflection over the 30 min interval.

Residual central force was then estimated from the relaxation modulus. These 30 min values are geometry-specific estimates derived from rectangular-bar testing, not direct clinical orthodontic forces, but they still serve as a useful comparative index of short-term force stability under standardized conditions (Figure 2).

### 3.3. FTIR Spectral Analysis

**Figure 3:**
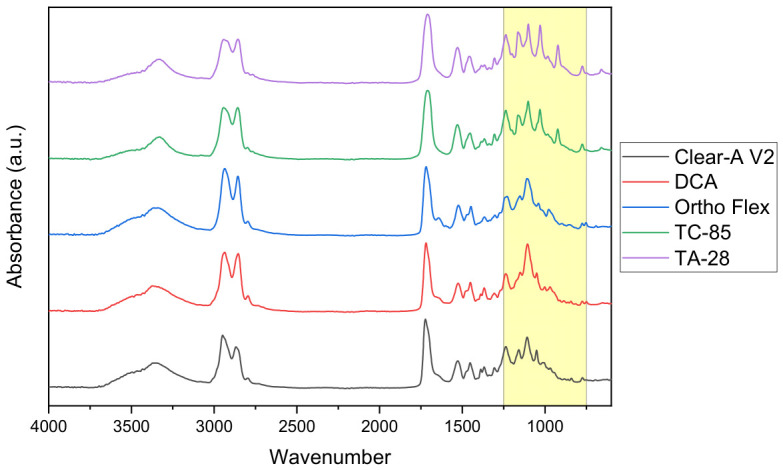
FTIR spectra of the different samples. The fingerprint region for acrylate and methacrylate (1250–750 cm^−1^) is shaded in yellow.

FTIR spectroscopy allowed the resins to be characterized chemically in qualitative terms, even without access to their disclosed formulations. The spectra averaged from three replicates appear in Figure 3, and most samples carried features consistent with polyurethane–methacrylate systems. Prominent absorption bands emerged at 3371 cm^−1^ (N-H stretching) and 1523 cm^−1^ (secondary amine), suggesting the presence of urethane linkages. Strong aliphatic C-H symmetric and asymmetric stretching peaks were present at 2953 and 2857 cm^−1^, respectively. A sharp peak at 1720 cm^−1^ confirmed the C=O stretching typical of ester and urethane bonds, while double bands at 1634 and 1620 cm^−1^ were attributed to C=C stretching within the methacrylate backbone [17].

Across all samples, the spectra pointed to crosslinked acrylate or methacrylate monomers, as expected for light-cured dental resins. The main differences lay in the shape and intensity of the peaks within the fingerprint region (1250–750 cm^−1^). Here TA-28 and TC-85 stood apart, showing a markedly different peak pattern from the other resins, most likely reflecting differences in their acrylate-to-methacrylate ratios or co-monomer structures. These subtle distinctions were enough to sort the resins into broadly similar chemical profiles [18]. Because the spectra were purely qualitative, they cannot pin down degree of conversion or crosslink density, so any link to the mechanical behavior must be interpreted with caution. Even so, the distinctive fingerprint of TA-28 and TC-85 fits well with their comparatively low final relaxation modulus.

## 4. Discussion

Taken together, our results confirm that direct-printed aligner resins are best treated as material-specific photopolymer systems, not as a single, interchangeable alternative to thermoformed foils. Direct printing is often presented as a promising route because it can trim workflow steps, sidestep thermoforming-related distortion, and allow tighter control over appliance geometry and thickness [5,19]. Yet recent reviews are equally quick to point out how heterogeneous the current evidence is, spanning different resin chemistries, printing workflows, post-processing protocols, aging conditions, and testing methods [5,6]. Laboratory findings should therefore be read within the exact material-and-processing combination that produced them, rather than generalized to every 3D-printed aligner.

What matters mechanically for an aligner is not simply how stiff it is at the outset. A clinically useful material has to combine enough rigidity to drive programmed tooth movement, enough flexibility for insertion and removal, enough elastic recovery to hold its shape, and enough control over viscoelastic behavior to keep force from decaying too quickly during wear. Striking that balance is genuinely hard, because polymeric aligner materials are inherently time-dependent. Our findings reinforce the idea that static flexural testing, taken alone, describes aligner performance only partially, especially when the clinical goal is to sustain force under constant deformation.

The literature on thermoformed aligners offers a helpful point of comparison. Thermoforming can change the thickness, morphology, optical properties, and mechanical response of thermoplastic foils, and simulated aging together with intraoral exposure can shift stiffness and force transmission still further [20,21]. Stress relaxation, too, has been documented repeatedly in thermoplastic aligner materials under simulated oral conditions, with material-specific differences in both the size and the speed of force decay [7,8,22]. Long-term studies add that this relaxation is not confined to the first hours after insertion but can continue across clinically meaningful intervals [8,23].

Direct printing reshapes this problem without eliminating it. Unlike thermoformed foils, printed aligners rely on crosslinked photopolymer networks whose final properties hinge on monomer and oligomer composition, degree of conversion, post-curing, and build strategy [5,24]. In principle, this opens up a wider design space, since resin chemistry and appliance geometry can be tuned together. In practice, that same freedom brings new sources of variability. Temperature-responsive and shape-memory photopolymers are especially attractive for orthodontics because they can improve adaptation, recoverability, and handling at mouth temperature [14,25]. The catch is that these very thermomechanical features may also heighten viscous participation at 37 °C, which is exactly why stress-relaxation behavior is essential for deciding whether a material can maintain effective loads over time.

Clinically, our results argue that static and dynamic mechanical tests are complementary rather than interchangeable. Static bending captures load-bearing behavior within a defined displacement window; stress relaxation captures the ability to hold stiffness and force under the sustained deformation that occurs throughout treatment. The distinction matters because a material can absorb a great deal of mechanical work while loading yet still shed a large share of that force during the hold phase, undermining tooth movement. Material selection for aligner fabrication should therefore not lean on flexural modulus or maximum flexural stress alone. Relaxation ratio and residual-force estimates are best reported alongside the static parameters, so that the full material phenotype comes into view [8,22].

Residual-force estimation offers a useful bridge between bench testing and appliance design, provided it is read with care. Beam theory can turn a relaxation modulus into a geometry-scaled force estimate for standardized specimens, which supports comparison under controlled conditions [7]. A finished aligner, however, generates a complex three-dimensional force system that depends on local thickness, trimline design, tooth morphology, undercut engagement, attachments, staging, and intraoral aging. Residual force calculated from rectangular bars is thus best viewed as a comparative index between materials, not as a direct estimate of clinical orthodontic force, a distinction that guards against mistaking bench-top rankings for clinical recommendations.

The absolute values we obtained sit comfortably within the range already reported for directly printed aligner resins. Shirey et al. measured elastic moduli of roughly 38 to 431 MPa for direct-print resins [15], and dynamic mechanical analysis of TC-85 has put its storage modulus near 714 MPa at 37 °C [14]; the flexural and relaxation moduli found here (approximately 320 to 1150 MPa) share the same order of magnitude, with the stiffest resin, DCA, at the upper end, in keeping with its high glass-transition temperature and dimensional stability. The comparatively low apparent stiffness of TC-85 and TA-28 fits their low glass-transition temperature (about 42 °C for TC-85), which leaves these shape-memory resins close to their transition at intraoral temperature and drives a marked drop in storage modulus between 30 and 45 °C [12,14]; a greater flexibility of TC-85 among printed materials has likewise been reported after water storage [26]. Our estimated residual forces (0.06 to 1.9 N) are of the same order as the static force of about 0.12 N reported for TC-85 aligners after repeated loading and the 0.3 to 2.7 N range measured in three-point bending [14], which supports reading them as biologically plausible comparative indices rather than clinical force values. Finally, the maximum flexural stresses recorded here (12 to 25 MPa) fall below the flexural strengths of 55–67 MPa reported for shape-memory aligner specimens [13], simply because our protocol logged stress within a fixed 1 mm displacement window rather than at fracture; this difference in endpoint is worth keeping in mind when comparing absolute values across studies.

The chemical characterization carried out here supports the idea that mechanical heterogeneity among direct-printed aligner materials is tied to differences in polymer network structure. Because most commercial formulations are proprietary, the conventional route of classifying materials by disclosed composition is usually closed off. FTIR spectroscopy can still reveal broad functional-group patterns and fingerprint differences between resins, even if qualitative normalized spectra cannot quantify the degree of conversion, crosslink density, residual monomer content, or molecular mobility. Those parameters carry clinical weight, since molecular architecture and conversion govern stiffness, elastic recovery, water uptake, and viscous dissipation [5]. Work on orthodontic and dental photopolymers reinforces the point, showing that polymerization kinetics, UV exposure, and post-curing conditions can shape final properties considerably [17,18]. In our own data, the distinctive fingerprint of TA-28 and TC-85 in the 1250–750 cm^−1^ region coincided with their pronounced stress relaxation and low final relaxation modulus, while the spectrally more similar, stiffer resins held on to a larger fraction of their initial stiffness; this association remains descriptive only, because qualitative FTIR cannot establish the underlying network parameters.

Post-processing, then, is a central methodological issue in directly printed aligner research. The final mechanical behavior of a printed resin is set not only by its nominal commercial formulation but also by what happens after printing. Well-controlled post-curing can strengthen interlayer bonding and improve surface characteristics, degree of conversion, and flexural properties, whereas insufficient or inconsistent curing can leave a less stable network behind [24,27]. Environmental exposure matters just as much: water absorption, thermal cycling, enzymatic activity, and routine hygiene can all speed up stress relaxation or creep [7,28]. Future studies would do well to pair immediate post-curing tests with hydrothermal and intraoral aging protocols.

Manufacturing parameters likewise call for standardization. Printing orientation has produced inconsistent effects from one study to the next, probably because its influence is entangled with resin type, aligner geometry, and thickness [4,13,29]. Post-polymerization time and temperature, similarly, can move flexural strength, hardness, and dimensional stability [15,30]. These are not secondary technical footnotes; they are part of the material system itself. Any meaningful comparison across direct-printed aligner studies will depend on transparent reporting of printer, layer height, orientation, exposure settings, post-curing device, curing duration, temperature, and atmosphere.

For this reason, direct printing and thermoforming are better compared as competing manufacturing systems than ranked in a simple hierarchy. Thermoformed materials are subject to sheet thinning, thermal history, and aging-related mechanical changes [20,31]. Direct-printed materials may escape some of those limitations, but they bring their own resin- and process-dependent variability. The useful question, then, is not whether direct printing is universally better than thermoforming, but whether a particular printed material, made under a defined protocol, delivers the desired balance of stiffness, flexibility, elastic recovery, force persistence, biocompatibility, and dimensional stability for a given orthodontic indication.

Safety and chemical stability belong in the same conversation as mechanical performance. Direct-printed aligner resins can release measurable leachable compounds, and degree of conversion is tightly linked to both mechanical behavior and biocompatibility [32]. Alongside this, concerns have surfaced about microplastic release, surface degradation, and polymer aging in clear aligner systems [33,34,35]. None of this should be read as proof of categorical clinical risk for direct-printed aligners, but it does make the case for combined mechanical, chemical, and biological evaluation. Strong mechanical performance means little if a material cannot also show adequate conversion, chemical stability, and resistance to aging [17,36].

Recent work strengthens the case for this integrated framework. Oyonarte et al. compared thermoformed and direct-printed materials after prolonged immersion in water at 37 °C and found that direct-printed systems can show material-specific thermo-hydric and viscoelastic responses [37]. Puchert et al. reached a similar conclusion, reporting that water storage alters the mechanical properties of 3D-printed aligner materials and underscoring the value of aging protocols that go beyond immediate post-curing tests [26]. Full-aligner studies have started to measure force expression directly, through expansion mechanics and mandibular incisor retraction models that pit direct-printed against thermoformed appliances [38,39]. And short-term intraoral exposure studies of chemical and surface stability remind us that clinical translation hinges not only on initial mechanical performance but also on how the surface and chemistry behave after wear [40]. Collectively, these studies point toward a future evaluation of direct-printed aligners that weaves together static testing, stress relaxation, hydrothermal aging, full-aligner force measurements, and chemical stability assessment.

### Limitations

Several limitations temper these findings. First, we used standardized rectangular specimens rather than full-aligner geometries, so the results cannot reproduce the multiaxial deformation, tooth-aligner contact mechanics, attachment engagement, or insertion–removal cycles that occur clinically. Second, stress relaxation was probed at a single fixed deflection and over a short 30 min window; longer relaxation protocols, cyclic loading, thermal cycling, water aging, and exposure to cleaning agents will be needed to approximate the mouth more faithfully. Third, specimens were printed and post-cured according to manufacturer-recommended protocols, which mirrors realistic clinical workflows but does not isolate the individual effects of build orientation, layer thickness, post-curing duration, or post-curing atmosphere. In addition, the thin aligner-like geometry, the 20:1 span-to-thickness ratio, and the imposed 1 mm deflection together produce a large deflection-to-thickness ratio; under such conditions, the small-deflection Euler–Bernoulli relations we applied return apparent flexural values rather than ISO-standardized moduli [16], so absolute stiffness values should be compared cautiously with studies using standard coupons, even though the relative comparisons among resins tested under an identical geometry remain valid. Finally, the FTIR characterization was qualitative and cannot quantify degree of conversion, crosslink density, residual monomer content, or leachable compounds. The present findings are therefore best read as material-level evidence that awaits validation in full-aligner biomechanical models and in clinical studies.

## 5. Conclusions

Direct-printed aligner resins differed markedly, and in a material-dependent way, in both static flexural behavior and short-term stress relaxation. Under the present conditions at 37 °C, DCA offered the most balanced profile, pairing high static stiffness, strong load-bearing response, and high unloading stiffness with comparatively slow stress relaxation. Clear-A V2 combined favorable static performance with intermediate dynamic stability, whereas TC-85 absorbed considerable work yet softened pronouncedly over time. Ortho Flex was weaker statically but held a moderate relative share of its stiffness, while TA-28 presented the least favorable overall profile.

The broader lesson is that static flexural testing alone cannot fully characterize directly printed aligner materials. Relaxation ratio and geometry-scaled residual force deserve to sit alongside the conventional static parameters whenever resin-specific performance is compared. Translating any of this into clinical practice, however, will require further validation with full-aligner geometries, longer aging, cyclic loading, and standardized printing and post-curing.

## Figures and Tables

**Table 1:** Static three-point bending properties of direct 3D-printed aligner resins tested at 37 °C (mean ± SD, *n* = 5).

Property (Unit)	Ortho Flex	Clear-A V2	DCA	TA-28	TC-85	*p* (KW)
σmax (MPa)	11.77 ± 0.42	22.01 ± 0.80	25.07 ± 0.90	12.91 ± 0.47	21.58 ± 0.78	2.0 × 10^−4^
E_f (MPa)	397.7 ± 14.5	732.1 ± 26.6	818.6 ± 29.9	320.9 ± 11.7	537.6 ± 19.8	1.2 × 10^−4^

**Table 2:** Stress-relaxation parameters of the same 3D-printed aligner resins during a 30 min hold at 1 mm mid-span deflection at 37 °C (mean ± SD, *n* = 5).

Property (Unit)	Ortho Flex	Clear-A V2	DCA	TA-28	TC-85	*p* (KW)
E_0_ (MPa)	368.9 ± 8.7	580.8 ± 13.6	1149.7 ± 27.0	353.2 ± 8.3	325.7 ± 7.6	1.4 × 10^−4^
E_30_ (MPa)	137.3 ± 3.3	192.3 ± 4.7	777.0 ± 18.9	23.5 ± 0.6	35.3 ± 0.9	1.2 × 10^−4^
Relaxation (%)	62.77 ± 0.06	66.89 ± 0.05	32.42 ± 0.10	93.33 ± 0.01	89.16 ± 0.02	1.2 × 10^−4^
RR	0.372 ± 0.001	0.331 ± 0.001	0.676 ± 0.001	0.067 ± 0.000	0.108 ± 0.000	1.2 × 10^−4^
Residual force (N)	0.34 ± 0.01	0.48 ± 0.01	1.94 ± 0.05	0.06 ± 0.00	0.09 ± 0.00	1.2 × 10^−4^

## Data Availability

The original contributions presented in this study are included in the article. Further inquiries can be directed to the corresponding author.
